# Utilization of Wheat Bran Acid Hydrolysate by *Rhodotorula mucilaginosa* Y-MG1 for Microbial Lipid Production as Feedstock for Biodiesel Synthesis

**DOI:** 10.1155/2019/3213521

**Published:** 2019-12-12

**Authors:** Ines Ayadi, Hafedh Belghith, Ali Gargouri, Mohamed Guerfali

**Affiliations:** Laboratory of Molecular Biotechnology of Eukaryotes, LMBE, Centre of Biotechnology of Sfax, P.O. Box 1177, TN-3038 Sfax, Tunisia

## Abstract

The lignocellulosic hydrolysate was used as the fermentation feedstock of *Rhodotorula mucilaginosa* Y-MG1 for the production of microbial lipids as the potential raw material for biodiesel synthesis. On synthetic media and under nitrogen-limiting condition, the Y-MG1 strain produces 2.13 g/L of lipids corresponding to 32.7% of lipid content. This strain was able to assimilate a wide range of substrates, especially C5 and C6 sugars as well as glycerol and sucrose. Fatty acid composition shows a divergence depending on the nature of used carbon source with a predominance of oleic acid or linoleic acid. An effective hydrolysis process, based on diluted acid treatment, was established for providing the maximum of fermentable sugars from different characterized lignocellulosic wastes. The highest yield of reducing sugars (56.6 g/L) could be achieved when wheat bran was used as the raw material. Hydrolysate detoxification step was not required in this study since the Y-MG1 strain was shown to grow and produce lipids in the presence of inhibitors and without the addition of external elements. Operating by controlled fed-batch fermentation yielded a dry biomass and oil yield of up to 11 g/L and 38.7% (w/w), respectively. The relative fatty acid composition showed the presence of increased levels of monounsaturated (66.8%) and saturated (23.4%) fatty acids in lipids of Y-MG1 grown on wheat bran. The predictive determination of biodiesel properties suggests that this oil may effectively be used for biodiesel production.

## 1. Introduction

The search of new alternatives for fossil fuels has become necessary, especially after the worldwide energy crisis. Biodiesel, one of the promising forms of bioenergy, has attracted attention in recent years for its nontoxic, biodegradable, renewable, and ecologic properties [1]. Biodiesel is produced mainly by the transesterification of vegetable oils, whereas vegetable oil production presents many constraints related to the dependence on climatic conditions, seasons, availability of arable land, and manpower. Microbial oil, known as single cell oil (SCO), is one of the most promising renewable oils which can replace the vegetable oils since they have a similar fatty acid composition [2]. Microbial lipids are produced by oleaginous microorganisms that synthetize and accumulate lipids more than 20% (w/w) of their total dry biomass weight and can reach up to 70% in specific conditions [3]. Under nitrogen-limiting conditions, the excess of carbon sources (such as glucose) will be used not for biomass production but rather for the synthesis of lipids, mainly triglycerides, which are used mainly for biodiesel synthesis [4]. Microbial oil is produced by several microorganisms such as yeast, microalgae, bacteria, and fungi. In fact, yeasts share several advantages for lipid production compared to other oleaginous microorganisms. They have a short duplication times, a unicellular form, and are very easy to grow in controlled and intensive systems [2]. In addition, one of the most important characteristics of yeast resides in its ability to use a large variety of raw materials for lipid production such as industrial wastewater [5], municipal organic wastes [6], crude glycerol [7], and lignocellulosic acid hydrolysate [8, 9]. The most known genera of oleaginous yeast are *Candida*, *Cryptococcus, Lipomyces, Rhodotorula*, *Trichosporon*, and *Yarrowia* [10]. However, the high cost of SCO production is the major obstacle for its larger commercialization. This limitation is mainly related to the fermentation processes and the nature of raw material used [1]. The use of inexpensive raw materials as a substrate for lipid production can help avoid this constraint. In addition, the selection of more interesting and robust yeast strains (able to grow in extreme conditions and the presence of a high level of pollutants and inhibitors) can be considered as a promising way [11]. Consequently, several studies have emerged showing the cost-effectiveness of microbial lipid production processes using low-cost fermentation substrates and more efficient yeast strains [1]. Lignocellulosic materials are the most interesting feedstock as a natural, available, and renewable resource. Thus, the massive amounts of lignocellulosic biomass (LB) can be converted into several high value products such as biofuels, value added fine chemicals, and bioactive molecules [12]. LB is composed of three major units, cellulose about 40–50%, hemicellulose about 20–30%, and lignin about 10–25%. Cellulose, the major component of LB, is a linear chain of D-glucose molecules while hemicellulose, the second component, is composed of repeated polymers of pentoses and hexoses [12]. Lignin has the role of glue by assembling between and around the cellulose and hemicellulose offering rigidity to the lignocellulosic structure. Therefore, the pretreatment is considered a crucial step to break down the lignin linkages and to retrieve cellulose and hemicellulose which are the source of sugars that will be used by microorganisms. Actually, many biotechnological hydrolysis processes were developed to generate cost-effective fermentable sugars such as physical treatment; physicochemical treatment including steam explosion, acid-based hydrolysis, alkali-based hydrolysis [1], hydrothermal hydrolysis [13], and ozonolysis [12]; and the biological treatments based on enzymatic hydrolysis [1]. Acid hydrolysis is the most studied and used technique among various pretreatment categories. It is a fast, inexpensive, and widely used method for producing sugars from lignocellulosic biomass [1]. Among several types of acids tested until now, dilute sulfuric acid was the most commonly applied catalysts [14]. Various lignocellulose derived toxins, including furan derivatives (furfural and 5-hydroxymethylfurfural (HMF)), organic acids (acetic acid, formic acid, and ferulic acid), and lignin derivatives (vanillin, 4-hydroxybenzaldehyde, guaiacol, and phenol), are generated during acid pretreatment [15]. These toxic compounds exert an inhibitory effect on the growth of microorganisms and the cost of their removal will significantly reduce the profitability of the bioconversion process [16]. In order to increase the bioconversion rate of lignocellulosic biomass, several detoxification processes have been developed to eliminate and reduce toxic compounds derived from the hydrolysis step [17]. The overliming using the calcium hydroxide and the combination of overliming and adsorption using the activated charcoal or the amberlite resin are the most studied of chemical and physical detoxification methods [1, 9]. These techniques have many disadvantages such as the massive freshwater usage and wastewater generation, loss of the fine lignocellulose particles and fermentative sugars, and the incomplete removal of inhibitors [15]. On the other hand, the biological detoxification can be considered a promising option; however, this technique requires the addition of specific enzymes and microorganisms [15]. All of these detoxification treatments lead to increase the cost of the bioconversion process, so other alternatives must be found to enhance the lipid yield production [18]. Several previous works have studied many kinds of lignocellulosic materials with different hydrolysis processes for lipids production such as corn stalk, bagasse, corncob, and wheat straw [8, 17–19]. The rate of lipid production depends on the nature of LB as a supplier of sugars and the potential of microorganisms that will convert sugars into energy molecules. Using acid hydrolysis, *Rhodotorula glutinis* CGMCC 2.703 and *R. glutinis* CBS strains produce 5.5 g/L (36.4%) and 1.4 g/L (11.86%) when cultured on corncob hydrolysate and wheat straw hydrolysate [8, 20].

In the present report, the newly isolated oleaginous yeast, *Rhodotorula mucilaginosa* Y-MG1, was evaluated for its potential to use lignocellulosic acid hydrolysate as a carbon source for microbial lipid production as potential raw material of biodiesel. Different lignocellulosic wastes were tested for fermentable sugars yields releasing after acid hydrolysis treatment. Without the need for detoxification, operating by controlled fed-batch fermentation of wheat bran hydrolysate allows increasing significantly the amount of microbial lipid produced. The current study provides an alternative way of bioconversion of lignocellulosic wastes to produce renewable energetic molecules.

## 2. Materials and Methods

### 2.1. Chemicals and Oleaginous Yeast Strain

Glucose, xylose, arabinose, sucrose, lactose, and glycerol were supplied from Bio Basic Canada Inc. Furfural, 5-hydroxymethyl furfural (HMF), and 3,5-dinitrosalycilic acid were purchased from Sigma Chemical Co. (St. Louis, MO, USA). Agroindustrial by-products (wheat bran and sugarcane bagasse) were kindly provided by a local food processing industry (White-rose group, Sfax, Tunisia). Almond shell, date palm leaves, and *Posidonia oceanica* balls were collected locally. All lignocellulosic residues are conserved at 4°C until use.

The new oleaginous yeast strain Y-MG1, isolated from rotten fruit, was identified in our previous work based on its internal transcribed spacer (ITS) sequence [21]. Y-MG1 strain was identified as being *Rhodotorula mucilaginosa* and was submitted in the National Strains Collection of Centre of Biotechnology of Sfax, CBS, Tunisia, under the accession number CTM-30138. The ITS sequence of Y-MG1 was also submitted to GenBank under the accession number ID: KX347596.1. This strain was stored at −80°C in sterilized glycerol-enriched solution containing 2% (w/v) glucose, 1% (w/v) yeast extract, 1% (w/v) bacto-peptone, and 20% (w/w) glycerol.

### 2.2. Acid Hydrolysis of Lignocellulosic Residues

Various lignocellulosic by-products were used as the raw material for fermentable sugars production. Wastes were firstly mechanically milled using electric chopper (rotary shear shredder) to obtain a particle size smaller than 2 mm and then washed with sodium hydroxide solution at low concentration (100 mM), followed by intensive washing with deionized water before drying for 48 h at 50°C [22]. Prepared lignocellulosic residues were then suspended and stirred at room temperature in 2% (v/v) dilute sulfuric acid solution at a solids loading of 10% (w/v). Thereafter, mixtures were autoclaved under different programs: P1: temperature 115°C, time 60 min, and pressure 0.6 bar; P2: temperature 121°C, time 60 min, and pressure 1.1 bar; P3: temperature 133°C, time 20 min, and pressure 2.1 bar; and P4: temperature 121°C, time 20 min, and pressure 1.1 bar. Hydrolysates were then filtered on Whatman No. 1 filter paper, neutralized with a 10N NaOH solution until pH 6.0, and used for the determination of reducing sugars.

### 2.3. Preculture Media

Y-MG1 strain was grown in 250 mL Erlenmeyer flasks containing 50 mL of preculture medium with the following composition: glucose, 20 g/L; bacto-peptone, 10 g/L; and yeast extract, 10 g/L. The pH of the media was adjusted to 6.0, and prior to inoculation, the preculture broth was sterilized at 121°C for 20 min. The incubation of the preculture was conducted at 30°C and under 180 rpm of agitation for 18 h. Cultures for lipid production were inoculated with 5% (v/v) of the preculture media corresponding to 0.2 unit of OD at 600 nm.

### 2.4. Single Cell Oil Production on Synthetic Media

Cultivation of the yeast using synthetic media for lipid production was in 0.5 L Erlenmeyer flasks containing 100 mL of culture. The synthetic medium composition was the same as described in our previous work [23] containing (in g/L) Na_2_HPO_4_ 2.5, KH_2_PO_4_ 7, (NH_4_)_2_SO_4_ 0.5, yeast extract 0.5, MgSO_4_·7H_2_O 1.5, CaCl_2_·2H_2_O 0.2, ZnSO_4_·7H_2_O 0.01, MnSO_4_·H_2_O 0.07, FeSO_4_·7H_2_O 0.01, and CuSO_4_ 0.0001. The initial pH of yeast cultures was adjusted to 6.0, and sugar concentration in all the experiments was set at 40 g/L providing a C/N ratio equal to 100. The carbon sources were commercial sugars such as glucose, xylose (alone or in mixtures), arabinose, sucrose, lactose, and polyol (glycerol). Culture samples were withdrawn regularly, and at the end of fermentation, the yeast cells were centrifuged at 3000 ×g for 5 min and harvested for the biomass determination and lipid extraction. All experiments were done at least thrice to ensure reproducibility.

### 2.5. Cultivation in Bioreactor

Microbial lipids were produced by the oleaginous yeast Y-MG1 using fed-batch fermentation in 7.0 L stirred tank bioreactor (Infors, AG GH-4103 Bottmingen, Switzerland) with a 4.0 L of initial working volume. The bioreactor (dished bottom glass-jacketed reactor) was equipped with an instrument for measurement and/or control of agitation, temperature, pH, and dissolved oxygen concentration. The cultivation temperature was fixed at 30°C and culture pH was kept constant at pH 6.0 by automatic addition of KOH (1 M) and H_3_PO_4_ (1 M). Agitation and aeration rates were maintained at 400 rpm and 1.5 vvm, respectively. Silicone (426 R) antifoam was added to control foam production. The fed-batch fermentation was conducted on wheat bran acid hydrolysate containing 60 g/L of reducing sugars and 2.4 g/L of nitrogen and supplemented with a mineral solution containing MgSO_4_·7H_2_O, 1.5 g/L; CaCl_2_·2H_2_O, 0.2 g/L; FeSO_4_·7H_2_O, 10 mg/L; MnSO_4_·H_2_O, 0.07 mg/L; ZnSO_4_·7H_2_O, 10 mg/L; and CuSO_4_, 0.1 mg/L. A volume of 160 mL of concentrated hydrolysate (using a rotary evaporator) with an initial reducing sugars concentration of 280 g/L was used to feed the culture when the residual sugars level in the medium became equal to or less than 10 g/L. During fermentation, samples were withdrawn regularly for biomass determination, sugars consumption, and lipid quantification.

### 2.6. Lipids Extraction

Microbial lipids were extracted according to Dey and Maiti [10] with some modifications. Briefly, a 100 mL culture sample was centrifuged at 3000 ×g for 10 min; the cell pellet was washed twice with 50 mL of distilled water and dried for 24 h at 105°C. 300 mg of dry matter was mixed with 10 mL of HCl (4 M) and incubated at 70°C for 2 h. The acid-hydrolyzed cells were stirred in 20 mL of chloroform: methanol mixture (1 : 1) at room temperature for 3 h, followed by centrifugation at 1500 ×g for 10 min to separate the aqueous upper phase and organic lower phase. The organic phase (containing lipids) was recovered and transferred to another glass tube. Finally, 20% (v/v) of 9 g/L NaCl was added to remove any residual moisture, solvent was removed by evaporation, and the total lipid was weighed. Lipid content was expressed as the percentage (w/w) of the extract on the dry biomass.

### 2.7. Analytical Methods

#### 2.7.1. Biochemical Composition of Lignocellulosic Residues

The dry weight of lignocellulosic residues was determined by oven drying 1.0 g of each sample at 105°C until a constant weight. Mineral components were determined as ash after incineration of an aliquot of the material at 550°C according to the NREL protocol, LAP-001 [24]. Acid-insoluble lignin content (Klason Lignin) was determined by a modified version of the method described in TAPPI T222, for acid-insoluble lignin in wood and pulp. Acid-insoluble lignin content (Klason Lignin) was determined by a modified version of the method described in TAPPI T222, for acid-insoluble lignin in wood and pulp. As lignin is insoluble in sulfuric acid, so fibers were stirred firstly in 75% H_2_SO_4_ for 2 hours and then the acid solution was diluted to reach 3% of concentration. Subsequently, the solution was heated to boiling with refluxing for 4 h. Finally, the residue was filtered and washed with 500 ml of water and dried at 100°C, representing lignin fraction. Acid soluble lignin (%) was quantified from sulfuric acid hydrolysates by measuring the absorbance at 205 nm.

The cellulose content of various lignocellulosic residues was determined using a Fibertec machine (Tecator, 1010 Heat extractor). Briefly, 1.0 g of fiber was firstly treated with 100 mL of a boiling sulfuric acid (1.25%) for 30 min followed by 100 mL of a boiling sodium hydroxide (1.25%). After 30 min, the residue was filtered and washed 3 times with hot water before washing with acetone (for 2 min). In the end, the residue was dried at 105°C to a constant weight and then incinerated at 550°C for 2 hours. The cellulose fraction represents the difference of residue mass before and after incineration.

The holocellulose content was determined according to the standard of American Society for Testing and Materials (ASTM) D 1104-56 (Reapproved 1978): 2 g of fibers was treated with 0.2 mL of glacial acetic acid and 1.0 g of sodium chlorite (NaClO_2_) at 75°C for 5 hours (adding the same mixture each hour). After cooling the mixture to 10°C, the residue was filtered and washed with 500 mL of water and then dried at 100°C. Hemicellulose content is the difference between holocellulose and cellulose.

#### 2.7.2. Reducing Sugars, Nitrogen, and Cell Mass Determination

Reducing sugars were quantified by the 3,5-dinitrosalicylic acid (DNS) method [25]. Nitrogen concentrations in lignocellulosic acid hydrolysate and in culture samples were analyzed by the Kjeldahl method [26]. To determine the amount of cell biomass, 10 mL of cell suspension sample was centrifuged at 3000 ×g for 5 min. The cell pellet was then washed twice with distilled water and dried in a preweighed glass tube at 105°C for 24 h, and the final mass was expressed as dry cell weight.

#### 2.7.3. Lignocellulosic Hydrolysate Compositions

Wheat bran hydrolysate composition including sugars (glucose and xylose) and inhibitors (5-hydroxymethylfurfural (HMF), furfural, and acetic acids) was determined using HPLC (Agilent Technology 1260 Infinity, refractive index detector RID, Agilent, USA) equipped with a Bio-Rad Aminex HPX-87H column. Sulfuric acid at 5 mM was used as a mobile phase with a flow rate of 0.6 mL/min, and at the column, temperature was at 65°C [9]. All compounds analyzed are used as standard at a concentration of 1 g/L.

#### 2.7.4. Neutral Lipid Composition and Biodiesel Characterization

Fatty acid composition of extracted microbial lipid was determined using GC/MS analysis. Firstly, the crude lipid was converted to fatty acids methyl esters (FAME) as follows: 100 mg of lipids was solubilized in 2 mL of hexane and supplemented with 0.2 mL of 2 M methanolic KOH solution. Subsequently, the mixture was incubated for 15 min at room temperature. Finally, the upper phase was recovered [27] and then analyzed by gas chromatographic system coupled to a series 5975 B Inert MSD Mass-Selective Detector (Agilent Technologies, France). 2 *μ*L of FAME aliquot was injected employing an HP-5MS Phenyl Methyl Siloxane capillary column (30 m × 250 *μ*m × 0.25 *μ*m nominal). Helium was used as a carrier gas with a constant flow (1 mL/min). The temperatures of the injector and detector were 250 and 240°C, respectively. The temperature program is as follows: 120°C for 5 min, an increase of 3°C/min to 180°C, an increase of 10°C/min to 220°C, and 220°C for 31 min. Data were evaluated using the NIST Mass Spectral Search Program.

The biodiesel is produced with a transesterification reaction where the TAGs are converted to fatty acid methyl esters with acidic or alkaline catalysts in the presence of alcohol and generating of glycerol as a by-product. The biodiesel characterization was conducted using the Biodiesel-Analyser software (Ver. 1.1, 2013). This analytical software was designed to predict biodiesel fuel properties (bioprospecting of FAME profile) of any oil feedstock profile determined by gas-chromatography [28]. The biodiesel characterization prediction is based on the fatty acid methyl ester profile of the oil feedstock. The estimation of the various properties from the fatty acid methyl ester profile (FAME) and the structure of the relevant fatty acids are based on 12 equations available in the study of Talebi et al. [28].

## 3. Results and Discussion

### 3.1. Oleaginous Yeast Selection

The main limitation of the marketing of microbial lipids is largely related to the high cost of raw materials converted to biodiesel. Lignocellulosic residues used as feedstock for lipids production can partially resolve this constraint. However, this type of bioconversion process requires more efficient microorganisms able to assimilate and convert efficiently the C6 and mainly C5 sugars into lipids [29]. In our previous work, using the qualitative fluorometric technique (based on Nile red staining), twelve new yeast strains with an interesting lipogenic character were selected [21]. One of them, the strain Y-MG1, characterized by simultaneous use of glucose and xylose as carbon sources, was also able to accumulate intracellular lipids when it was cultivated on nondetoxified lignocellulosic acid hydrolysate showing a good resistance against the inhibitors. The Y-MG1 strain was identified, using molecular technique, as being *Rhodotorula mucilaginosa* [21]. The yeast *R. mucilaginosa* was previously described for its ability to produce interesting metabolites such as microbial lipids, carotenoids, and enzymes [30, 31].

### 3.2. Lipid Accumulation on a Synthetic Medium by *R. mucilaginosa* Y-MG1

**Figure 1:**
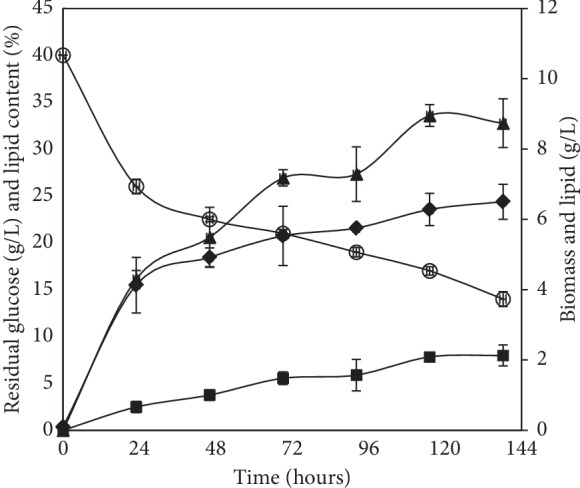
Time courses of biomass (♦), lipid production (■), lipid content (▲), and glucose consumption (○) of *R. mucilaginosa* Y-MG1 cultivated in Erlenmeyer flask and using 40 g/L of glucose as a carbon source.

It is necessary to evaluate the capacity of lipids production by oleaginous yeast on synthetic media before using it for lignocellulosic hydrolysate as a substrate. To this end, batch culture in shake flasks was carried out on a nitrogen-limited medium with 40 g/L of glucose as a carbon source (C/N ratio about 100). The time courses of cell growth, lipid production, and sugar consumption are illustrated in Figure 1. During the first 24 hours of culture, exponential growth was observed and accompanied by consumption of about 15 g/L of glucose. The biomass gradually increased up to a maximum of 6.5 g/L after 144 h of cultivation. For lipid accumulation, very slight production appeared in the first 24 hours and increased during the stationary phase to reach 2.13 g/L corresponding to 32.76% of lipid content. In such nitrogen-limited conditions, the quickly depletion of nitrogen element in the growth medium causes deceleration of cell growth, and consequently, the assimilated carbon will be directed to synthesizing secondary metabolites such as fatty acids [4]. Cultivated under nitrogen-limited condition and in the presence of a high concentration of the carbon source (60 g/L of glucose corresponding to C/N ratio of 150), TJY15a, another yeast strain of *R. mucilaginosa*, produced 30.4% of lipid content [32]. Likewise, when it was cultivated on glycerol (50 g/L) as a unique carbon source, *R. mucilaginosa* produced around 3.1 g/L after 72 h of culture [33].

### 3.3. Carbon Source Effect on Lipids Accumulation and Fatty Acid Composition of *R. mucilaginosa* Y-MG1

Besides glucose, other substances like disaccharides, polyols, and hydrophobic substrates could also serve as substrates for single cell oil production by oleaginous yeasts [34]. In addition, the fatty acids profile of accumulated lipids has been shown to be largely dependent on the nature of the carbon source used [23] which will determine the potential application of these lipids. To this end, the effect of carbon sources on cell growth, lipids accumulation, and relative fatty acids composition of *R. mucilaginosa* Y-MG1 was investigated and the results are shown in Table 1.

All substrates were added at an equal amount of carbon and supplemented with 0.16 g/L of nitrogen source (yeast extract/(NH_4_)_2_SO_4_) to reach a C/N ratio of 100. This strain is able to use a large panel of substrates, especially, C6 and C5 sugars as well as glycerol and sucrose. However, lactose and arabinose are not suitable substrates for lipids accumulation; only 15.7 and 11.7% of lipid content were observed, respectively. The highest amount of lipid was detected when Y-MG1 was cultivated on glucose (1.84 g/L corresponding to 27.65% of lipid content) followed by sucrose and xylose (1.74 g/L and 1.53 g/L, respectively). There is no significant improvement in lipid production when glucose and xylose were mixed. This result was similar to that obtained by Sha [35] where the production of biomass and lipid was similar when glucose and the mixture of glucose/xylose were used as a carbon source by four oleaginous yeasts (*L. lipofer*, *L. starkeyi*, *R. glutinis*, and *Y. lipolytica*) [35]. Similarly, in the study of Hu et al. [36], they have tested many combination ratios of glucose and xylose for *Trichosporon cutaneum* AS 2.571 cultivation and lipids production. No enhancement in lipid rate was detected compared with cultivation on glucose. Lipid production was improved only in 3-liter bioreactor batch fermentation using glucose/xylose 2 : 1 combination [36]. On the other hand, the use of the glucose/xylose combination as a carbon source significantly improves the lipids production by other oleaginous yeast strains such as *C. viswanathii* Y-E4 and *T. cutaneum* CTM-30125 (4.28 g/L and 4.58 g/L, respectively) [9, 23]. Also, we noticed that Y-MG1 was able to assimilate and synthesize lipids when grown on glycerol (lipid content of 27.08%). We shall notice that glycerol is a potential waste of agri-food and biorefinery industries and its conversion into fatty acids would be an economically profitable alternative [1]. In addition, the ability of the strain Y-MG1 to assimilate sucrose is an interesting feature as many oleaginous strains cannot metabolize it efficiently. For example, the wild strain of *Y. lipolytica* is unable to grow on sucrose [37]. However, the expression of the gene encoding *S. cerevisiae* invertase in this yeast made it possible to circumvent this difficulty [38]. At the industrial scale, the Y-MG1 strain could grow efficiently on inexpensive residues which are considered as sucrose-rich substrates such as molasses. Lipids extracted from *R. mucilaginosa* Y-MG1 were transmethylated and analyzed by GC/MS (Table 1). The fatty acid (FA) composition shows a divergence according to the nature of the carbon source used. The lipids produced on glucose, xylose, and sucrose were essentially composed of oleic acid, a major compound, followed by palmitic acid and a relatively low amount of linoleic acid (maximum of 14.5%). This FA composition profile is similar to that of vegetable oil, suggesting that the lipid produced by this yeast could be a potential candidate for biodiesel production. However, linoleic acid becomes the predominant compound in lipids extracted from glycerol, arabinose, and glycose/xylose cultures (>33%). Ahmad et al. [39] demonstrated that FA profile was slightly affected by the nature of the carbon sources used in the culture media of *R. mucilaginosa* with a predominance of C18 : 1 followed by the C16 : 0 and C18 : 2 [39].

### 3.4. Biochemical Characterization of Lignocellulosic Wastes

In order to produce microbial lipids via an efficient bioprocess, five types of lignocellulosic materials were used (wheat bran, sugarcane bagasse, date palm leaf, *Posidonia* balls, and almond shell) as feedstock. Generally, lignocellulosic biomass is composed of three main elements: cellulose, hemicellulose, and lignin. The relative proportion of these components depends mainly on the origin of the material [40]. For this reason, a biochemical characterization step was carried out for the different residues based on the normalized process (Table 2). Cellulose fraction is the major compound in all lignocellulosic wastes (39.72% to 55.76%) except in wheat bran where hemicellulose is the major compound (52.7%). The high content of cellulose and hemicelluloses (sources of fermentable sugars) is of great interest in the exploitation of waste lignocellulosic material. Likewise, the study of Merali et al. [13] carried out on wheat bran shows that the major compound is hemicellulose with about 51% [13]. The compositions of sugarcane bagasse, date palm leaves, and *Posidonia* pellets are in agreement with those published in the literature [41–43] except for almond shell where hemicellulose is the major compound, with 35% [44]. For all the studied wastes, the amount of lignin was relatively low and varies between 7% and 19.94%. Generally, the low amount of lignin facilitates the hydrolysis of lignocellulosic materials and enhances the recovery yield of sugars [45]. In addition, during the hydrolysis process, the high amount of lignin increases the formation of phenolic derivatives that affect microbial proliferation [18].

### 3.5. Selection of Lignocellulosic By-Product

**Figure 2:**
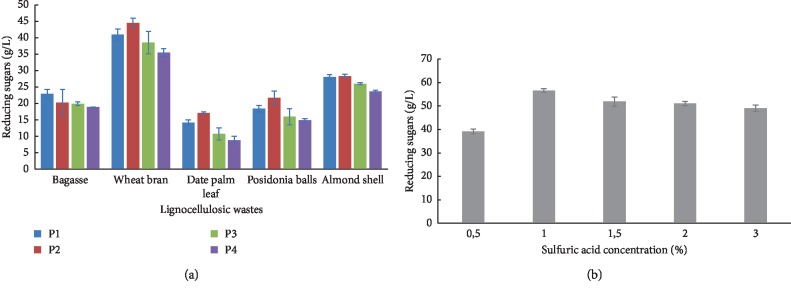
(a) The effect of autoclave programs on reducing sugars released from different lignocellulosic wastes. (b) The effect of sulfuric acid concentrations on reducing sugars released from wheat bran. P1: temperature 115°C, time 60 min, and pressure 0.6 bar; P2: temperature 121°C, time 60 min, and pressure 1.1 bar; P3: temperature 133°C, time 20 min, and pressure 2.1 bar; P4: temperature 121°C, time 20 min, and pressure 1.1 bar.

The exploitation process of LB requires three steps: the preparation of plant material called pretreatment step, the hydrolysis step that serves to release the monosaccharides, and the third step is the bioconversion that uses microorganisms [46]. In the first step, acid treatment is the chemical processes mostly used for LB degradation [47–49]. To this end, the different LB has undergone a heat treatment coupled with sulfuric acid hydrolysis. The sulfuric acid concentration was set at 2% while temperature, pressure, and time of treatment were varied. The result of LB hydrolysis is illustrated in Figure 2(a).

Wheat bran shows the maximum of reducing sugars under all autoclaving programs and allows 44.54 g/L of released sugar with the program 2 (121°C, 60 min, 1.1 bar). This is due to its richness in hemicellulose which is mainly affected by acid treatment and also due to its low lignin content [17]. However, sugarcane bagasse, date palm leaf, *Posidonia* balls, and almond shell do not exceed 28 g/L of liberated reducing sugars. In previous works, rice straw and corncob LB treated with 1.5% sulfuric acid at 121°C for 90 min and 2.5% sulfuric acid at 135°C for 60 min release 35.2 g/L and 45.7 g/L of sugars, respectively [47, 49]. The sugarcane bagasse produces 21.38 g/L of sugars when it was hydrolyzed with 2.5% HCl, at 121°C for 45 min [48]. Based on the amount of released sugars, wheat bran was selected as the feedstock for microbial lipid production by the Y-MG1 strain. Moreover, wheat bran is a cheap and abundant source of dietary fiber which is produced as a by-product in several processes of wheat treatment in milling industries [50]. Worldwide consumption of wheat, according to the World Agricultural Supply and Demand Estimates (WASDE), has been estimated to 652.18 million tons for the year 2010 (WASDE, 2010). One million tons of wheat can produce up to 0.25 million tons of wheat bran. This agriculture residue has relatively diverse applications in food, feed, health, and fermentation industries due to its richness in carbohydrates (mostly fibers), protein, and fats [51].

On the other hand, the acid concentration is also one of the important factors in the chemical hydrolysis of the lignocellulosic materials. For this purpose, different concentrations of sulfuric acid (0.5–3%) were applied for wheat bran hydrolysis using the best program (P2) (Figure 2(b)). At 1% of sulfuric acid, wheat bran releases the maximum amount of sugars reaching a concentration of 56.61 g/L. The increase in the concentration of sulfuric acids is not accompanied by an increase in the amount of sugars released. In general, the use of high acid concentrations can generate a significant amount of by-products such as HMF, furfural, and acetic acid that are unfavorable for the growth of microorganisms [48]. In addition, using low concentrations of acid reduce the environmental impact of the bioconversion process.

### 3.6. Lipid Production in Bioreactor by *R. mucilaginosa* Y-MG1 Using Wheat Bran Hydrolysate

**Figure 3:**
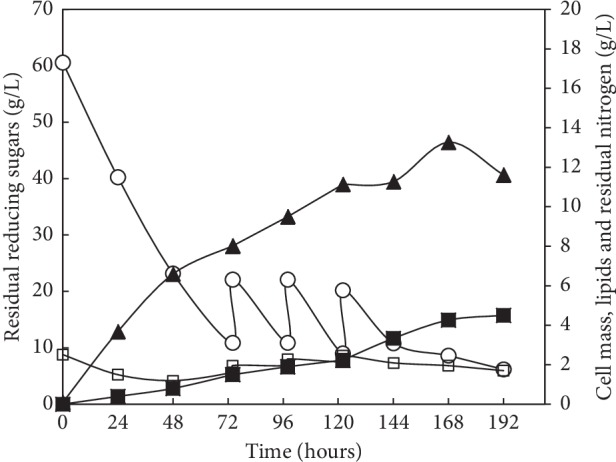
Time courses of biomass (▲), lipid production (■), reducing sugars (○), and total nitrogen (□) consumption of *R. mucilaginosa* Y-MG1 cultivated on acid wheat bran hydrolysate under feed-batch fermentation.

Carbohydrate, nitrogen, and inhibitors composition of acid wheat bran hydrolysate was determined before using this raw material as a carbon source for microbial lipid production by Y-MG1 strain. The composition based on HPLC analysis was as follows: glucose 23.7 g/L, xylose 19.1 g/L, HMF 0.69 g/L, furfural 0.07 g/L, and acetic acid 0.73 g/L. Total reducing sugars and nitrogen were about 56 g/L and 2.4 g/L, respectively. During wheat bran hydrolysate fermentation, the addition of external nitrogen source was not necessary to avoid the decrease of the C/N ratio and its consequent negative effect on lipid synthesis. In addition, the presence of inhibitors such as acetic acid (the most abundant aliphatic acid formed by the hydrolysis of acetyl groups of hemicellulose), furfural, and HMF (the degradation by-products of pentoses and hexoses, respectively) can limit microbial growth. Several studies operate through detoxification techniques to increase the efficiency of the bioconversion of carbohydrates [16]. Our study was conducted to set up a cost-effective bioconversion process. To do this, we chose not to include detoxification treatments. The yeast strain *R. mucilaginosa* Y-MG1 was previously tested for its capacity of growing and producing lipid on acid-WBH without detoxification and in the presence of inhibitors [21]. Under batch fermentation, Y-MG1 produces about 2.3 g/L of lipid corresponding to 20.4% of lipid content [21]. Productivity and lipid yields can be enhanced using the fed-batch fermentation. This culture mode allows also avoiding problems of mixing and heat transfer due to the high concentration of sugars in the culture medium [52]. To this end, *R. mucilaginosa* Y-MG1 was cultivated on nondetoxified WBH (initial reducing sugars concentration was about 60.5 g/L) supplemented only by an elements solution (ZnSO_4_, MnSO_4_, FeSO_4_, and CuSO_4_) and at fixed pH 6.0. The evolution of biomass and lipid production, as well as the consumption of sugars and nitrogen, was determined every 24 hours (Figure 3). We observed two fermentation phases. The first phase extends from the beginning of the culture until 74 hours. This phase was characterized by exponential growth and allows reaching 8.0 g/L of biomass and 1.5 g/L of lipid accompanied by a consumption of 47.6 g/L of sugars and 1.0 g/L of nitrogen. The second phase (feeding phase) begins when the concentration of residual sugars exceeds the minimum threshold of 10 g/L. Three successive feedings were performed with each feed containing 11.2 g/L of sugars and 0.33 g/L of nitrogen. This allowed the biomass to grow gradually to reach a maximum of 13.3 g/L after 168 h before declining at the end of the culture. The progressive addition of WBH allows producing 4.5 g/L of lipid. The conversion efficiency of sugars into lipid (*Y*_L/S_) has evolved from 0.03 g/g (during the first phase) to 0.12 g/g (after feeding phase). However, biomass productivity (*Y*_X/S_) was clearly superior during the first phase (0.16 g/g). This is explained by the consumption of sugars in favor of cell growth in the first phase, whereas, in the second phase, a metabolized sugar was directed toward lipid synthesis. The same result was observed when the oleaginous yeast, *Cryptococcus* sp. SM5S05, was cultivated on corncob acid hydrolysate under fed-batch fermentation [52] giving a final production rate of lipids of 4.5 g/L.

Exploiting new abundant LB and reducing the use of costly nutritional additives for microbial growth are a challenge to set up a cost-effective process for microbial lipid production. Different reports have exploited lignocellulosic material as raw material for the production of lipids by oleaginous yeasts (Table 3). The process of microbial lipid production was related to several factors affecting its productivity such as culture conditions, yeast species, the nature of raw material, and nitrogen source. Compared with other yeast of *Rhodotorula* genus, the lipid content of Y-MG1 (38.79%) was higher than that of *R. glutinis* (25%) [18] and almost similar to that of *R. glutinis CGMCC* 2.703 (36.4%) and *R. mucilaginosa* (36.91%) when grown on nondetoxified acid corncobs and corn stalk hydrolysates [8, 19]. Cultivation of *C. starkeyi* on bagasse hydrolysate under different culture modes had resulted in lipid yields not exceeding 28% [17]. In addition, *C. tropicalis and T. cutaneum* CX1 cultivated on palm empty fruit and corn stover hydrolysate, respectively, produce only 1.6 and 3.1 g/L of lipids using batch bioreactor culture [53, 54]. However, the lipid yield of Y-MG1 was lower than that of *R. mucilaginosa* (6.64 g/L) using the nondetoxified wheat straw hydrolysate [19]. All of this shows that the nondetoxified acid wheat bran hydrolysate is another potential lignocellulosic source that could be used as feedstock for microbial lipid production.

### 3.7. Fatty Acid Composition and Biodiesel Characterization

Fatty acid (FA) profile of lipids extracted from *R. mucilaginosa* Y-MG1 after growth on nondetoxified WBH was determined by GC-MS analysis.  Table 4 shows that oleic acid C18 : 1 was the major compound of lipids (66.12%) followed by palmitic acid C16 : 0 (11.94%) and stearic acid C18 : 0 (11.06%). Nevertheless, the polyunsaturated fatty acids did not exceed 8%. This lipid composition is highly similar to *L. starkeyi* and *T. cutaneum* when grown on a corncob and barley hulls acid hydrolysates, respectively [9, 55]. This composition, largely identical to that of many vegetable oils, was considered as ideal for biodiesel synthesis [56]. Indeed, the quality of biodiesel depends on the fatty acid composition of lipids, which must be rich in saturated and monounsaturated FAs and parallel poor in polyunsaturated FA [56]. Methyl esters from monounsaturated FAs guarantee a good quality of biodiesel because they are in liquid form at room temperature and characterized by good flow properties, unlike methyl esters from polyunsaturated FAs which induce oxidation problems during storage [56]. The theoretical characterization of *R. mucilaginosa* biodiesel was performed using BiodieselAnalyser software [28]. Some biodiesel properties are shown in Table 3 with US standards (ASTM D-6751). The cetane number is an indicator of the ignition quality of a diesel fuel. It measures the readiness of the fuel to autoignite when injected into the engine [57]. The biodiesel of *R. mucilaginosa* shows a cetane number equal to 56.68, which conforms to the norm (≥47). The iodine value influences several important properties of biodiesel, such as the clogging point of the cold filter and the oxidation stability [58]. The maximum limit of the iodine value is 120 and the biodiesel of *R. mucilaginosa* complies with an index of 57.9. The kinematic viscosity of biodiesel is also an important parameter because it indicates the ability of the fuel to form deposits in the engine [58]. The biodiesel of *R. mucilaginosa* has a kinematic viscosity of 1.39 (mm^2^/s) which is close to the lower limit 1.6 (mm^2^/s). The biodiesel density of *R. mucilaginosa* is slightly below the minimum limit. In the applied domain, this limitation can be circumvented by mixing the *R. mucilaginosa* biodiesel with other denser products. We conclude that the lipids extracted from the yeast *R. mucilaginosa* can be considered as very suitable for the production of biodiesel.

## 4. Conclusion

In our current study, we have shown the feasibility of producing microbial oil from a renewable raw material as lignocellulosic wastes. The newly isolated oleaginous yeast, *R. mucilaginosa* Y-MG1, was able to utilize acid wheat bran hydrolysate for oil production without prior detoxification. Moreover, the addition of an external nitrogen source was not needed during wheat bran hydrolysate conversion, which is beneficial for the economics process. Fed-batch fermentation allowed a significant increase in the amount of microbial lipids produced by Y-MG1 strain. The resulting Y-MG1 lipids were finally considered as a promising feedstock for biodiesel production.

## Figures and Tables

**Table 1:** Biomass production and lipids accumulation by *R. mucilaginosa* Y-MG1 cultivated for 5 days on various carbon sources.

Carbon source	*X* ^a^ (g/L)	*L* ^b^ (g/L)	*Y* ^c^ (%, w/w)	Relative fatty acid content (%)				
				C16 : 0	C16 : 1	C18 : 0	C18 : 1	C18 : 2
Glucose	6.64 ± 0.55	1.84 ± 0.36	27.65 ± 3.13	15.20	1.40	6.30	58.30	14.50
Xylose	6.14 ± 0.14	1.53 ± 0.08	24.94 ± 1.89	25.51	0.84	5.71	50.06	0.11
Arabinose	4.99 ± 0.24	0.58 ± 0.01	11.73 ± 0.26	10.03	—	9.31	26.96	36.44
Sucrose	6.37 ± 0.22	1.74 ± 0.04	27.3 ± 0.34	15.44	0.66	9.25	68.45	3.09
Lactose	0.65 ± 0.01	0.1 ± 0.01	15.71 ± 3.03	ND	ND	ND	ND	ND
Glycerol	4.57 ± 0.68	1.19 ± 0.1	27.08 ± 6.32	14.69	0.94	13.11	25.52	40.69
Glucose/xylose (1 : 1)	6.25 ± 0.04	1.69 ± 0.07	27.15 ± 1.07	15.37	1.03	10.13	29.05	33.16
Glucose/xylose (2 : 1)	6.31 ± 0.41	1.71 ± 0.23	26.95 ± 1.87	14.48	1.38	9.90	25.67	40.53

^a^Biomass; ^b^lipid yields; ^c^lipid content.

**Table 2:** Biochemical composition of different lignocellulosic biomasses.

Raw material	Cellulose	Hemicellulose	Lignin	Ash	Other
Wheat bran	30.04 ± 0.08	52.7 ± 0.92	7 ± 0.76	6.19 ± 0.01	4.07
Sugarcane bagasse	52.45 ± 0.65	25.22 ± 1.73	17.12 ± 1.14	1.68 ± 0.01	3.53
Date palm leaf	39.72 ± 0.68	21.97 ± 0.71	16.05 ± 0.87	4.31 ± 0.05	17.95
Almond shell	55.76 ± 0.32	16.74 ± 0.5	10.29 ± 0.65	2.84 ± 0	14.36
Posidonia balls	52.12 ± 0.05	4.96 ± 0.97	19.94 ± 1.12	3.73 ± 0.29	19.25

**Table 3:** Lipid production from lignocellulosic biomass using oleaginous yeasts.

Strains	Lignocellulosic residues	Nitrogen source	Culture mode	*X* ^a^ (g/L)	*L* ^b^ (g/L)	*Y* ^c^ (%)	Reference
*C. tropicalis*	Palm empty fruit bunch	(NH_4_)_2_SO_4_	Batch bioreactor	6.40	1.60	25.00	[53]
*T. cutaneum CX1*	Corn stover hydrolysate	(NH_4_)_2_SO_4_	Batch bioreactor	10.20	3.10	30.40	[54]
*L. starkeyi*	Acid bagasse hydrolysate	(NH_4_)_2_SO_4_	Flask	9.30	2.50	26.90	[17]
			Batch bioreactor	9.60	2.51	26.10	
*R. glutinis CGMCC 2.703*	Corncobs hydrolysate	(NH_4_)_2_SO_4_	Continuous	11.5	3.14	27.30	[8]
			Batch bioreactor	15.27	5.50	36.40	
*R. glutinis*	Wheat straw hydrolysate	Yeast extract	Flask	13.80	3.50	25.00	[18]
*R. mucilaginosa*	Wheat straw hydrolysate	(NH_4_)_2_SO_4_	Flask	15.88	6.64	41.81	[19]
*R. mucilaginosa* Y-MG1	Corn stalk hydrolysate acid wheat bran hydrolysate	—	Fed-batch bioreactor	15.55	5.74	36.91	This work
				11.6	4.50	38.79	

^a^Biomass; ^b^lipid yields; ^c^lipid content.

**Table 4:** Fatty acid profile of *R. mucilaginosa* Y-MG1 lipid and corresponding biodiesel parameters.

Fatty acids	% (w/w)	Biodiesel characterization		
C16 : 0	11.94		Y-MG1 biodiesel	Standard
C16 : 1	0.72	Cetane number	56.68	≥47
C18 : 0	11.06	Iodine value	75.45	120 max
C18 : 1	66.12	Saponification value	199.55	—
C18 : 2	7.1	Kinematic viscosity (mm^2^ s^−1^)	1.39	1.6–6
C18 : 3	0.88	Density (g cm^3^)	0.86	0.87–0.89
C20 : 0	0.44			

## Data Availability

(i) Oleaginous yeast selection and microbial lipids production and quantification, (ii) lignocellulosic residues characterization and degradation using diluted acid treatment, and (iii) the corresponding results of bioreactor fed-batch fermentation and biodiesel synthesis and characterization data used to support the findings of this study are included within the article.
